# Correction: Empirical prediction of patent pledge financing of pharmaceutical enterprises—A case study in Jiangsu China

**DOI:** 10.1371/journal.pone.0333991

**Published:** 2025-10-07

**Authors:** Xiaojuan Zhao, Yunhua Liu

## Notice of Republication

This article was republished on September 16, 2025, to correct errors in the Supporting Information file that were incorrectly included in the originally published article. Please download this article again to view the correct version. The originally published, uncorrected article and the republished, corrected articles are provided here for reference.

## Supporting information

S1 File Originally published, uncorrected article. (PDF)

S2 File Republished, corrected article. (PDF)
